# Toll-like Receptor Expression in *Pelodiscus sinensis* Reveals Differential Responses after *Aeromonas hydrophila* Infection

**DOI:** 10.3390/genes15091230

**Published:** 2024-09-20

**Authors:** Yu Tian, Hui Zhang, Lingrui Ge, Zi’ao Wang, Pei Wang, Shuting Xiong, Xiaoqing Wang, Yazhou Hu

**Affiliations:** 1 College of Aquaculture, Hunan Agricultural University, Changsha 410128, China; tianyu_1998@foxmail.com (Y.T.); zhanghui98626@outlook.com (H.Z.); stxiong@hunau.edu (S.X.); wangxiao8258@126.com (X.W.); 2 Department of Animal Science and Technology, Hunan Biological and Electromechanical Polytechnic, Changsha 410127, China; gelingrui@hnbemc.edu.cn (L.G.); wangziao@hnbemc.edu.cn (Z.W.); 3 College of Biology and Environmental Sciences, Jishou University, Jishou 416000, China; wangpei0229@126.com

**Keywords:** *Pelodiscus sinensis*, toll-like receptors, bacterial infection, immune response, microorganism binding

## Abstract

**Background**: Toll-like receptor (TLR), as an important pattern recognition receptor, is a bridge between non-specific immunity and specific immunity, and plays a vital role in the disease resistance of aquatic animals. However, the function of TLR in *Pelodiscus sinensis* is still unclear. **Methods and Results**: The sequence characteristics and homology of three TLRs *(PsTLR2*, *PsTLR3* and *PsTLR5*) were determined in this investigation. Their annotation and orthologies were supported by phylogenetic analysis, functional domain prediction, and sequence similarity analysis. qPCR showed that the identified TLRs were expressed in all tissues, among the high expression of *PsTLR5* in the brain and liver and the high expression of *PsTLR2* and *PsTLR3* in the liver. *PsTLR2* mRNA expression increased 6.7-fold in the liver 12 h after *Aeromonas hydrophila* infection, while the mRNA expression of *PsTLR3* was down-regulated by 0.29 times in liver and 0.31 times in spleen. The mRNA expression of *PsTLR5* was significantly up-regulated in four immune tissues, and it was up-regulated by 122.8 times in the spleen after 72 h infection. Finally, the recombinant proteins of extracellular LRR domains of these three TLRs were obtained by prokaryotic expression technology, and the binding tests were performed to discover their ability of binding pathogenic microorganisms. Microbial binding test showed that rPsTLR2, rPsTLR3 and rPsTLR5 can combine *A. hydrophila*, *Edwardsiella tarda*, *Vibrio parahaemolyticus*, *Staphylococcus aureus*, *Streptococcus agalactiae* and *Candida albicans*, while rPsTLR3 can bind *A. hydrophila*, *E. tarda*, *V. parahaemolyticus* and *C. albicans*. **Conclusions**: Our findings suggested that TLRs may be crucial to turtles’ innate immune response against microbes.

## 1. Introduction

Innate immunity is the most common host defensive mechanism observed in a wide variety of multicellular organisms [1]. Innate immune recognition is dependent on a small number of germline-encoded receptors [2]. Pathogenicity related molecular patterns (PAMP) are among those that may be identified utilizing pattern recognition receptors (PRRs) [3]. Toll-like receptor (TLR) is a key component of PRRs that may identify invasive infection and initiate the body’s healing process.

TLRs are transmembrane proteins of type I that have an internal Toll/interleukin-1 receptor (TIR) domain, a transmembrane domain, and external leucine-rich repeat (LRR) domains [4]. The LRR domain recognizes pathogens, whereas the TIR domain transduces signals downstream. TLRs identify a wide range of PAMPs, including flagellin, lipopolysaccharide (LPS), bacterial DNA, and single- or double-stranded viral RNA [5]. TLRs transmit downstream signals to the cytoplasm after recognizing PAMPs, where they activate two important adaptor proteins, the myeloid differentiation main response gene (88) (MyD88) and the Toll-like receptor adaptor molecule 1 (TICAM1, also known as TRIF). Multiple cytokines, including interleukin (IL)-12, IL-8, tumor necrosis factor alpha (TNF-α), and interferon (IFN), have been induced by these proteins. TLRs with different ligand specificities regulate different signaling pathways [6,7,8].

To date, mammals have been found to harbor 13 different types of TLRs, the majority of which are found in the genomes of aquatic animals [9,10]. They are classified into six TLR families (TLR1, TLR3, TLR4, TLR5, TLR7, and TLR11) based on their naturally occurring ligand type and recognized sequence [11]. TLR2, a crucial component of the TLR1 subfamily, is capable of identifying lipids, carbohydrates, and proteomes from microbes [12]. The ability of this receptor to generate functional heterodimers with more than two other TLR types is unique among TLRs. In addition, TLR2 interacts with non-TLR molecules, which makes a wide range of PAMPs recognizable [13,14]. As a member of the TLR3 subgroup, TLR3 is capable of identifying nucleotide derivatives that come from bacteria or viruses [15]. It has been demonstrated that TLR5 in fish and mammals is able to detect the flagellin protein component of bacterial flagella and is in charge of flagellin-mediated NF-κB activation [16]. The TLRs of *Pelodiscus sinensis* are still largely unexplored at the molecular level, with whole genome sequencing being one example of this [17,18].

TLR is a member of PPRs that has the capacity to recognize invasive infections and to trigger and mobilize the immune system. *P. sinensis* is an ancient reptile with significant nutritional and economic value as well as market growth potential. However, because to inadequate breeding practices, a lack of knowledge about germplasm protection, and inappropriate treatment use, *P. sinensis*’ illnesses have gotten worse over time [19]. While immune control is advantageous due to its good effect and reduced pollution, it is unclear how *P. sinensis* resists sickness and how immune it is. It is of great significance to study TLRs in *P. sinensis*. The purpose of this study is (1) to clone the TLR2, TLR3, and TLR5 genes of *P. sinensis*.; (2) to examine the tissue expression profile of TLRs and the expression profile during bacterial infection; (3) to produce recombinant proteins of the extracellular LRR domains of the three TLRs genes and identify their capacity to bind to microorganisms. This study may increase the immune gene database of *P. sinensis*, provide a theoretical reference for the analysis of disease-resistance mechanisms of *P. sinensis* and the development of prevention and control measures targeting TLRs.

## 2. Materials and Methods

### 2.1. Animals

All turtles, weighing about 200 g (±50 g), were obtained from Hezhou Turtle Breeding Base (Changde, China). The turtles were temporarily kept in a 50 L feeding box in the laboratory at 28 °C (±2 °C) before the test and were fed commercial pellets twice a day for 14 days. The animal experiments were according to the rules of the Animal Care and Use Committee of Hunan Agricultural University (Changsha, China; Approval Code: 202004297; Approval Date: 9 February 2020).

#### 2.1.1. Bacterial Infection Experiment

*A. hydrophila*, isolated from livers of diseased turtles, was identified and preserved by our laboratory. The injection concentration of bacteria was determined by pre-experiment. The experimental group of turtles received an intraperitoneal injection of 100 μL of *A. hydrophila* at a concentration of 5 × 10^9^ CFU/mL, whereas the control group of turtles received an intraperitoneal injection of 100 μL PBS. Immune-related tissues of the liver, spleen, kidney, and intestine from each group (*n* = 3) were sampled post-injection at 12, 24, 48, and 72 h, respectively. The samples were quickly frozen with liquid nitrogen and then stored at −80 °C before RNA extraction.

#### 2.1.2. RNA Extraction and cDNA Synthesis

Total RNA was separated in accordance with FastPure Cell/Tissue Total RNA Isolation Kit (Vazyme, Nanjing, China) from the collected tissues of *P. sinensis* in accordance with the manufacturer’s instructions. The RNA specimen quality was measured by a ratio of A260: 280. The integrity of RNA was verified by the electrophoresis of 1.5% agarose. Reverse transcription of RNA into first-strand cDNA was performed by Thermo Scientific RevertAidRT (Thermo, Waltham, MA, USA). For real-time PCR analysis, qPCR (+gDNA wiper) (Vazyme, Nanjing, China) was synthesized with HiScriptII Q RT superMix to synthesize the first-strand cDNA. The cDNA template was stored at −20 °C for later use.

#### 2.1.3. Gene Cloning of TLR2, TLR3 and TLR5

The amplification primers of these three genes were designed based on the predicted sequences (XM_025185305.1; XM_014576247.2; XM_025180981.1) in the NCBI database (Table 1). PCR was carried out on aMyCycler (BioRad, Hercules, CA, USA) as follows: at 94 °C for five minutes; then at 94 °C for thirty seconds, at 56 °C for thirty seconds, and at 72 °C for 180 s for 35 cycles; and finally extended for ten minutes at 72 °C. The PCR was purified, ligated in pMD19T carrier (Takara, Kyoto, Japan), and then cloned into DH-5α. A commercial service (Tsingke, Changsha, China) sequenced positive clones.

#### 2.1.4. Bioinformation Analysis of TLR2, TLR3, and TLR5

Comparison of the nucleotide and amino acid sequences was performed with the BLAST application (http://blast.ncbi.nlm.nih.gov/Blast.cgi/, accessed on 20 March 2023), based on the analysis of the derived amino acid sequences (https://web.expasy.org/protparam/, accessed on 20 March 2023), SMART (http://smart.embl-heidelberg.de/, accessed on 20 March 2023), and PROSITE (https://prosite.expasy.org/, accessed on 20 March 2023). Three dimensional (3D) models were built with SWISSMODEL (www.swissmodel.expasy.org/, accessed on 14 April 2023) and PyMOL 2.5.7. You can reconstruct more than one sequence with Clustal Omega (https://www.ebi.ac.uk/Tools/msa/clustalo/, accessed on 14 April 2023) and GENEDOC. The phylogenetic tree was built by means of MEGA 7.0 based on neighborhood connection (NJ).

#### 2.1.5. Quantitative PCR (qPCR) Analysis

In order to evaluate the mRNA levels of 3 TLR genes after bacteria injected into various tissues, RT-qPCR was used to detect and quantify real-time PCR. The Master Mix of ChamQ Universal SYBR qPCR (Vazyme, Nanjing, China) on LightCycler 96 (Roche, Basel, CH) was applied. The qPCR reaction mix included 5 μL of 2 × SYBR Premix Ex Taq II, cDNA template of 1 μL, sense or inverse primer (10 μM) 0.4 μL, and ddH_2_O 3.2 μL at the end volume of 10 μL. Each specimen was subjected to 3 replications, and β-actin was used as the cDNA normalization inner reference gene. The PCR procedure is as follows: 95 °C for 60 s, 95 °C for 5 s, 60 °C for 30 s, and then proceed to Step 2, where the process is repeated 40 times. The RT-qPCR dissociation profile was analyzed to show no contamination of DNA. Two sets of specimens were treated thrice with cDNA from three distinct bioassays (*n* = 3) as well as negative controls. A 2^−ΔΔCT^ approach was employed to compute relative expression levels for each gene [20]. The difference of expression was determined by T-test, and the *P* was less than 0.05 indicating statistically significant. The primers used are listed in Table 1.

#### 2.1.6. Recombinant Protein Expression and Purification

The mRNA leucine-rich repeats (LRRs) (*PsTLR2*-LRRs, *PsTLR3*-LRRs, and *PsTLR5*-LRRs) were amplified by using the following primers (Table 1), after the amplification of the LRRs was performed with a restriction enzyme pCold-TF. After recombinant plasmid purification via sequencing, it was converted to *Escherichia coli* BL21. The non-insertion pCold-TF, which could express rTFHs, could be used as a negative control. Then, the bacteria containing the expression vector were cultivated under Luria–Bertani (LB) medium at 37 °C and added with ampicillin (1 mg/mL), till OD600 was 0.6~0.8. Finally, a final concentration of isopropanol β-D-1-Thiogalactopyranoside (IPTG) was added to induce protein expression. After incubating at 16 °C for 16 h, the cells were collected by centrifuging for 10 min at 6000 rpm. The cell beads were re-suspended in Lysis buffer and then lysed in an ice-bath for 30 min by ultrasonic (2/2, 70%). The His-tagged proteins in the supernatants were purified in accordance with the manufacturer’s protocol with His-tag Purification Resin (Beyotime, Shanghai, China), and the BCA Protein Test Kit (Sangon, Shanghai, China) was performed for detection of the eluted samples’ concentration.

#### 2.1.7. Microbial Binding Assay

Pathogenic bacteria from aquatic animals (*A. hydrophila*, *Edwardsiella tarda*, *Vibrio parahaemolyticus*, *Staphylococcus aureus*, *Streptococcus agalactiae*, and *Candida albicans*) were selected for overnight culture in LB/PDA and then re-suspended in PBS at a concentration of 1.0 × 10^7^ cells/mL. Approximately 100 μL of purified rTLR2-LRRs, rTLR3-LRRs, and rTLR5-LRRs (100 μg/mL) were added as negative control at room temperature. The cells were removed by centrifuging (6000 revolutions per minute for 5 min) and then cleaned with PBS 3 times. For the Western blot assay, the last bacterial balls were used, and the antibody (Abkine, CA, USA) was used as the first antibody.

#### 2.1.8. Western Blotting

Incubation of the cells using a 5 × SDS loading buffer was boiled for 10 min. The samples were then subjected to the SDS-PAGE gel and transferred onto the PVDF. The film was incubated for 10 min with QuickBlock™ blocking buffer (Beyotime, Shanghai, China), then incubated with a primary antigen at 4 °C, followed by three times of PBST, and incubated with a Horseradish Peroxidase (HRP)-conjugated goat anti-rabbit or mouse antibody (1: 5000) for 1 h at room temperature. Finally, the film was washed using PBST and processed (Beyotime, Shanghai, China) using ECL Western blotting detection reagents. A minimum of three tests were performed.

#### 2.1.9. Statistical Analysis

Using GraphPad Prism 7.0, the average ± SD of the 3 biological tests were shown. The *t*-test (Both Groups) evaluated a statistically significant outcome. A *p* value below 0.05 was regarded as significant. All experiments were individually repeated at least three times.

## 3. Results

### 3.1. Cloning and Sequence Analysis of TLR2, TLR3, and TLR5

**Figure 1:**
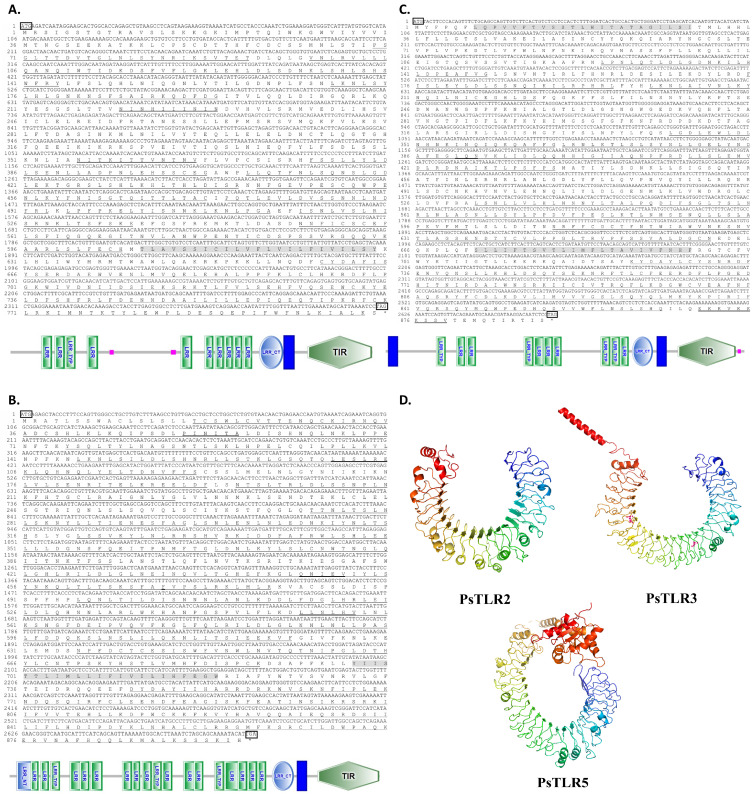
Sequence analysis of three TLR genes from *P. sinensis*. The top column contains the nucleotides of *PsTLR2* (**A**), *PsTLR3* (**B**), and *PsTLR5* (**C**), whereas the bottom column contains the inferred amino acid sequences. Gray shading indicates transmembrane domains. The start codon (ATG) and the stop codon (TAG) are marked with boxes. The C-terminal leucine-rich domain (LRR-CT) and the leucine-rich domain (LRR) are marked with single underlines. Overlapping regions are shown in bold underline, and the domains of TIR are marked with wavy lines. The double underline is used here to identify low complexity. The symbol ‘*’ is used here to identify terminator T. (**D**) Create a schematic model of the tertiary structure of TLRs proteins using SWISSMODEL and PyMOL display by applying SMART prediction.

The completed CDS of *P. sinensis* TLR2, TLR3, and TLR5 genes, named *PsTLR2*, *PsTLR3*, and *PsTLR5*, were successfully cloned and identified. Compared with the predicted sequences in the public database, they differ in sequence length and base composition (Supplementary Materials). As shown in Figure 1, the completed CDS of *PsTLR2* was 2412 bp in length and encoded 803 amino acids. The protein structure of *PsTLR2* was further predicted by the SMART analysis service. The deduced *PsTLR2* protein exhibited typical TLR domains, including two signal peptides, eleven leucine-rich repeat (LRR) motifs, a transmembrane region (TM), and a Toll/interleukin-1 receptor (TIR) domain. The CDS of *PsTLR3* was 2691 bp in length and encoded 896 amino acids. It contained nineteen LRR motifs, a TM, and a TIR domain. The *PsTLR5* gene was 2670 bp and encoded a protein with 889 amino acids, including twelve LRR motifs, two TMs, and a TIR domain.

3D models of *PsTLR2*, *PsTLR3*, and *PsTLR5* predicted by SWISS-MODEL indicated that the LRR domain of *PsTLR2*, *PsTLR3* and *PsTLR5* had a classic horseshoe structure, their transmembrane regions were composed of α helical, and the TIR domains included multiple serine/threonine residues. It can effectively recognize and bind to appropriate ligands by reason of its structural shape, which can start or control immune responses.

**Figure 2:**
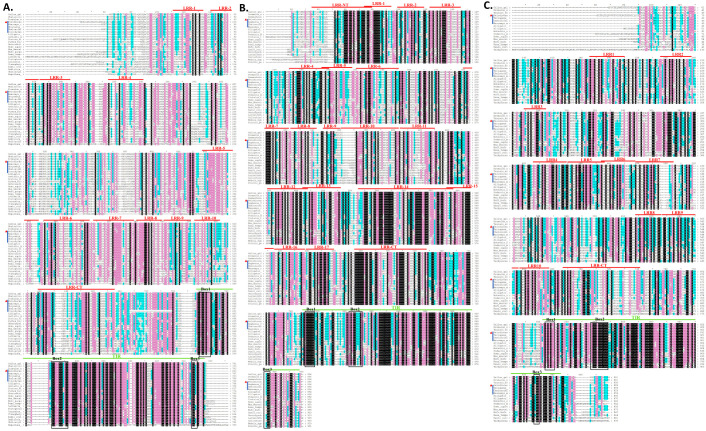

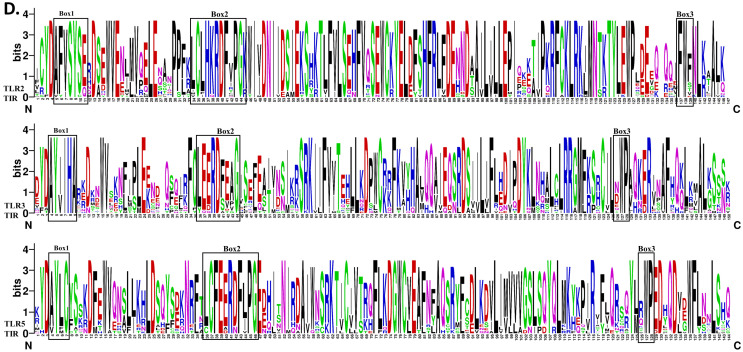
Multiple sequence alignment (**A**–**C**) and sequence logo (**D**) of amino acid sequence of TLRs in *P. sinensis* with TLRs from other species. The LRRs are shown in red line segments, and the TIR domain is shown in green line segments. Amino acid sequence is similarity color-coded: 100% in black, 70% in pink, 50% or more in blue, less than 50% in white. The three conserved boxes 1–3 are marked with black boxes. *PsTLRs* are marked with red triangles. Turtles are represented by blue vertical lines. The symbol ‘*’ is used here to identify interval.

BLASTP analysis indicated that the deduced amino acid sequences of three TLRs shared high similarities with other reported TLRs. *PsTLR2* showed 85.35% identity with MmTLR2 from *Mauremys mutica* (XP_044874668.1), *PsTLR3* closely matched the TLR3 homologs of *M. reevesii* (XP_039398302.1), and *PsTLR5* was similar to TLR5 proteins from *Trachemys scripta elegans* (XP_034620540.1). Multiple sequence alignment and sequence logo showed that the TIR domains of TLR genes in *P. sinensis* are highly conserved with other species. The TIR domain of TLR2 contains three box domains, namely Box1 (A-Y-), Box2 (L-RD-PG) and Box3 (-W-). The TIR domains of TLR3 and TLR5 also have three box domains (Figure 2).

**Figure 3:**
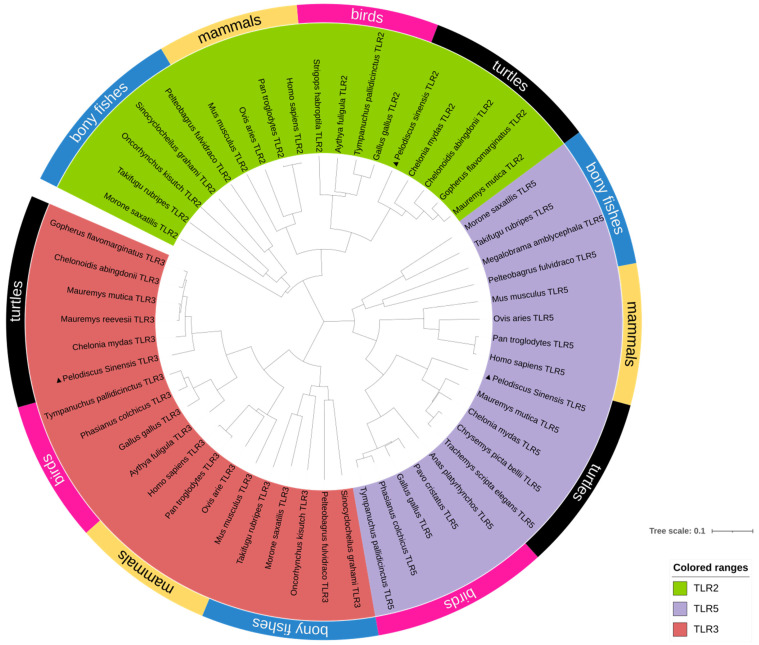
Phylogenetic tree of TLRs. The phylogenetic tree was built by means of a neighborhood joining approach with a bootstrapping of 1000 copies, as discussed in the literature and methods section. *PsTLRs* are marked with black triangles.

In order to determine the evolutionary relation between *PsTLR2*, *PsTLR3*, and *PsTLR5* with other species, including mammals, turtles, birds, and bony fish, a phylogenetic tree was established. As illustrated in Figure 3, all TLR2, TLR3, and TLR5 clustered into one clade each. *PsTLRs* first formed a clade with other tortoises, and then with mammals and birds. Bony fishes alone clustered into a clade. This finding is in line with the taxonomy findings, suggesting that TLR tended to be conserved during evolution. It is speculated that their function is relatively conservative.

### 3.2. Tissue Expression of TLR2, TLR3 and TLR5

**Figure 4:**
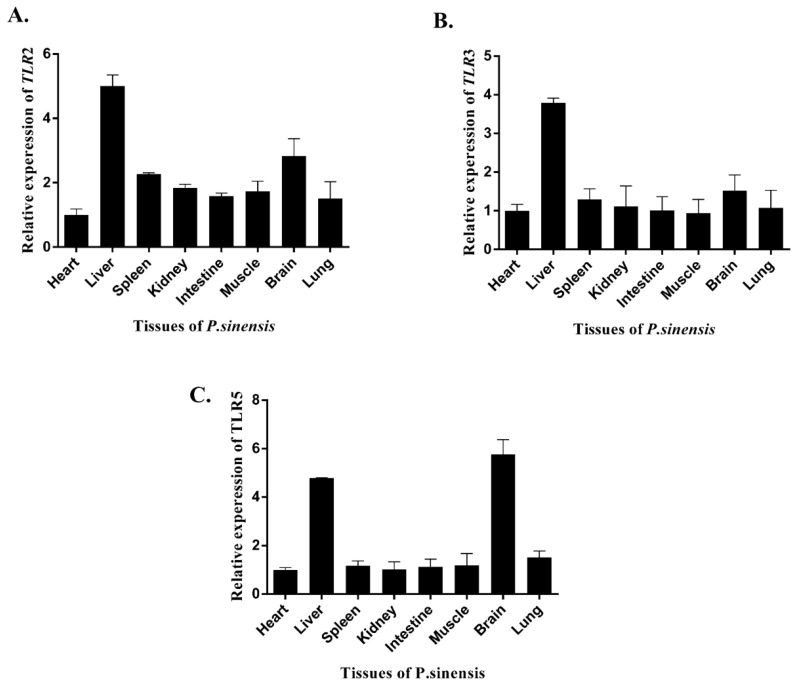
Healthy tissue-relative expression levels of PsTLR2 (**A**), PsTLR3 (**B**), and PsTLR5 (**C**) mRNA (heart, liver, spleen, kidney, intestine, muscle, brain, and lung).

RT-qPCR was employed to determine the *PsTLR2*, *PsTLR3*, and *PsTLR5* mRNA in different tissues of *P. sinensis*. *PsTLR2* was found to be ubiquitous in all of the examined tissues, moderately expressed in the brain, and highly expressed in the liver (Figure 4A). The expression of *PsTLR3* is high in the liver but low in the heart, brain, spleen, kidney, muscle, intestine, and lungs (Figure 4B). *PsTLR5* is found in liver, brain, and other tissues at low concentrations (Figure 4C).

### 3.3. Expression Profiles of TLR2, TLR3 and TLR5 Challenged by A. hydrophila

**Figure 5:**
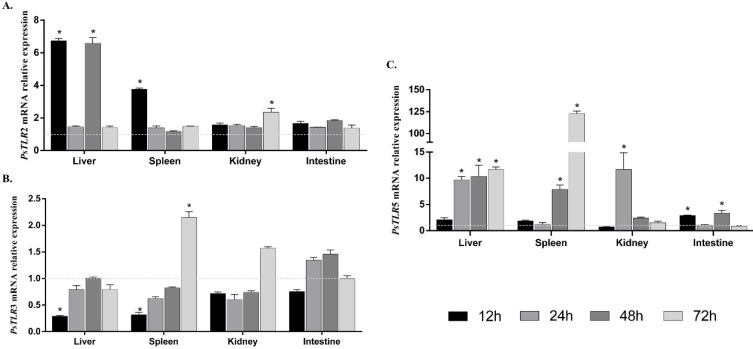
Temporal expression of three TLR genes after A. hydrophila infection. *PsTLR2* (**A**), *PsTLR3* (**B**), and *PsTLR5* (**C**) expression levels in the liver and kidney of *P. sinensis* after *A. hydrophila* infection were measured by RT-qPCR. Asterisks (*) represent significant difference (* *p* < 0.05).

It has been reported that TLR expression may be up-regulated following bacterial or flagellin stimulation. In order to preliminarily ascertain if TLRs are regulated in *A. hydrophila* infection, RT-qPCR was used to analyze the expression patterns of TLRs. The expression of *PsTLR2* in hepatic, splenic, and renal tissues was markedly elevated, with negligible alteration in the intestines (Figure 5A). In hepatic and splenic tissues, *PsTLR3* was first reduced, then rose, and reached a minimum at 12 h (Figure 5B). There was significant up-regulation of *PsTLR5* in four tissues. It was highest in the hepatic and splenic organs at 24 h and maximal in the kidneys at 12 h. Therefore, *PsTLR5* has an active reaction against the invading bacteria during the initial phase of the challenge (Figure 5C).

### 3.4. Prokaryotic Expression of TLR2, TLR3 and TLR5-LRR

**Figure 6:**
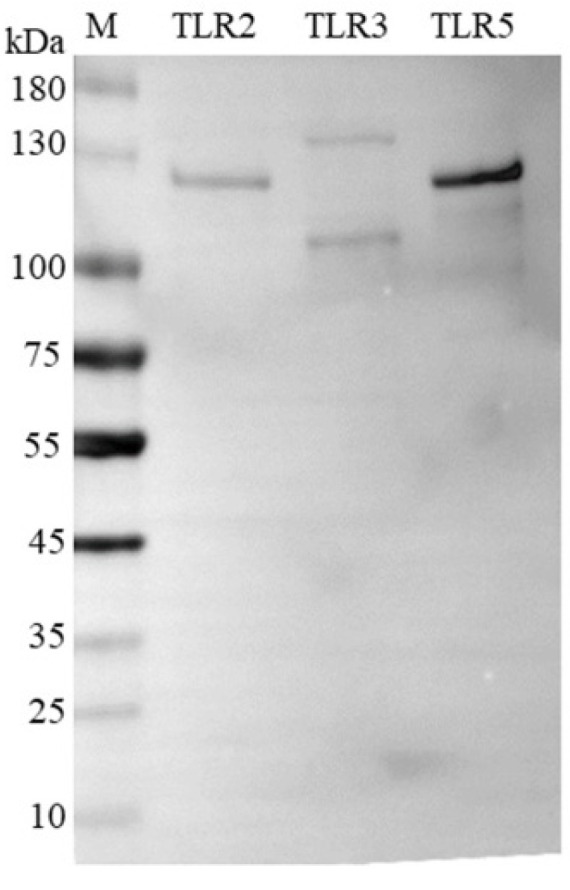
The recombinant proteins rPsTLR2-LRRs, rPsTLR3-LRRs, and rPsTLR5-LRRs were expressed and purified. Western-blot analysis of rPsTLR2-LRRs, rPsTLR3-LRRs, rPsTLR5-LRRs protein with the mouse-anti-6 × His-tag monoclonal antibody.

LRR domains were reported to recognize pathogens. To analyze the possible mechanism of *PsTLR2*, *PsTLR3*, and *PsTLR5* against bacteria, the ectodomain LRRs of *PsTLR2*, *PsTLR3*, and *PsTLR5* were recombinantly expressed in BL21. SDS-PAGE analysis showed that the rPsTLR2-LRRs, rPsTLR3-LRRs, and rPsTLR5-LRRs proteins were purified and resolved as a sharp band of ~120 kDa, which was close to the molecular weight of rPsTLR2-LRRs, rPsTLR3-LRRs, and rPsTLR5-LRRs. After Western blot assay using mouse-anti-6 × His-tag monoclonal antibody, a ~120 kDa band was observed, which was consistent with the predicted size of the rPsTLR2-LRRs, rPsTLR3-LRRs, and rPsTLR5-LRRs regions (Figure 6).

### 3.5. Binding Activities of TLR2, TLR3, and TLR5-LRR to Bacteria

**Figure 7:**
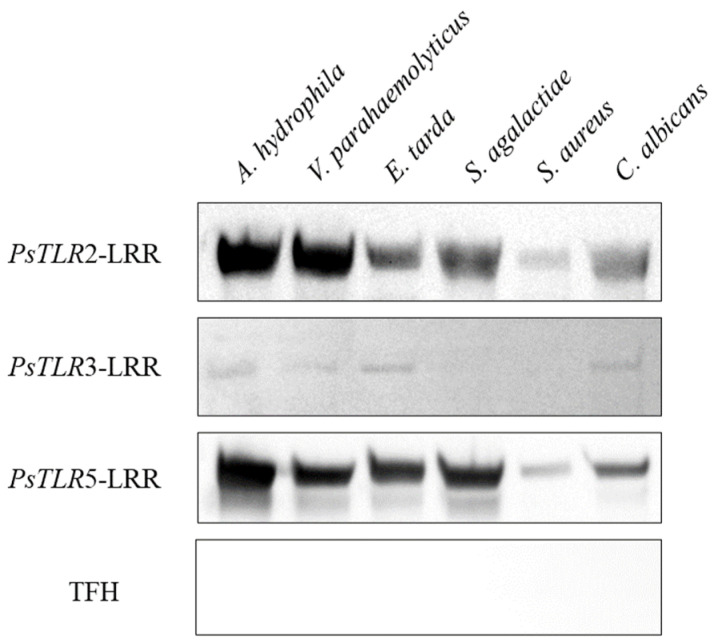
The microorganisms binding assay of rPsTLR2-LRRs, rPsTLR3-LRRs, rPsTLR5-LRRs and TFH recombinant protein. The binding ability was showed by Western blotting. TFH was used as negative control.

Then the purified rPsTLR2-LRRs, rPsTLR3-LRRs, and rPsTLR5-LRRs proteins were used to determine whether they could bind to bacteria. By Western blot assay (Figure 7), rPsTLR2-LRRs and rPsTLR5-LRRs were able to bind to all the tested bacteria, among which *A. hydrophila*, *V. parahaemolyticus*, *E. tarda*, and *S. agalactiae* showed the highest affinity, whereas rPsTLR3-LRR could only bind to *A. hydrophila*, *V. parahaemolyticus*, *E. tarda*, and *C. albicans*, and the rTFH protein (control) showed no binding activity to any bacteria.

## 4. Discussion

From the obtained results, the predicted sequences in the public database are not completely consistent with the experimental amplification results, indicating that the predicted sequences are not completely correct, and it is necessary for us to verify the predicted sequences in the public database. Nevertheless, these high-throughput sequencing-based data provide an important foundation for research. According to research, the structure of the TLR family is relatively conserved, typically featuring three domains: LRR domains, TM domain, and TIR domain. Three classical domains are present in each of the *PsTLR2*, *PsTLR3*, and *PsTLR5* genes, which is consistent with the traits of the TLR family.

The extracellular domain of the TLR family is a variable LRR domain that is important for ligand recognition and signal transduction and is involved in many physiological processes, including immune response and signal transduction [21,22,23]. Species differ in the number and kind of LRR domains [24]. But the TIR domain is rather conservative. It primarily contributes to TLR signal transmission and localization, which is essential for innate immunity. Through homotropic interactions, TIR enlists downstream TIR-containing signaling molecules to create a signaling complex [25,26]. Three conserved functional domains (Box1, Box2, and Box3) are found in the TIR domain. Box is a conserved amino acid sequence of the TIR domain, which is closely related to the signaling function. The boxes were discovered to be present in the TIR domain of each species of TLR by comparing their TIR domains [27]. Research has indicated that the TIR domain signal transduction is significantly influenced by Box1, that the dimerization and recruitment of adaptor proteins in the TIR domain are determined by the Bbloop site in the Box2 region, and that the functional intracellular region of TLR signal transduction, the Box3 region, is involved in directing the localization of the receptor [7,28]. The binding of TIR to proteins linked to signal transduction is facilitated by both Box1 and Box2. Through the results of multiple alignment of protein sequences and phylogenetic trees, we can see that the extracellular LRR domain of *PsTLR2*, *PsTLR3*, and *PsTLR5* was less conserved. The amino acid sequences of *PsTLR2* and *PsTLR5* each contain 11 LRR domains, similar to the number of LRR domains found in other turtles’ TLRs, such as *Gopherus flavomarginatus* TLR2 (XP_050802314.1), which has 11 LRR domains. *Mauremys reevesii* TLR2 (XP_039396763.1) has 10 LRR domains; *Chelonoidis abingdonii* TLR5 (XP_032643841.1) and *Terrapene triunguis* TLR5 (XP_024055424.2) have 12 LRR domains. The amino acid sequence of *PsTLR3* contains 19 LRR domains, which is less than the number of LRR domains of TLR3 in other turtles. For example, *G. flavomarginatus* TLR3 (XP_050803453.1) and *Gopherus evgoodei* TLR3 (XP_030420382.1) have 21 LRR domains. *PsTLR2* and *PsTLR3* have more LRR domains than other vertebrates; for example, *Megalobrama amblycephala* TLR2 (XP_048053737.1) and *Mus musculus* TLR2 (NP_036035.3) have only eight LRR domains. *Homo sapiens* TLR3 (NP_003255.1) and *M. musculus* TLR3 (NP_001344245.1) have 18 LRR domains. Therefore, in most vertebrates, different TLR genes have different leucine-repeat structures in the extracellular region, suggesting that they also play different recognition mechanisms in the process of pathogen recognition. The TIR domains of *PsTLR2*, *PsTLR3*, and *PsTLR5* were found to be highly conserved, and all of them contained three conserved box domains (box1, box2, and box3). This suggests that they stabilize efficient transduction signals, an important safeguard for the immune system against pathogens. This occurrence further demonstrated that the gene cloned was accurate.

The research revealed that *PcTLR2* was significantly expressed in the intestine of *Pseudosciaena crocea*, whereas *PcTLR3* was primarily expressed in the liver and then the intestine [29,30]. *Carassius auratus’*s liver, head kidney, and spleen all showed significant levels of *CaTLR2* expression [31]. *Micropterus salmoides’*s head kidney showed high expression of MsTLR3 [32]. TLR5m was widely distributed in all tissues in *Oncorhynchus mykiss*, whereas TLR5s was primarily expressed in the liver [33]. These findings suggest that distinct TLR isoforms within a species exhibit varying tissue selectivity, and that distinct species exhibit distinct TLR gene expression profiles. TLR mRNA is often highly expressed in immunological tissues like the gut, spleen, liver, and kidney in all species. According to certain studies, the spleen, head kidney, and blood of fish all take part in the immune response, suggesting that the intestine, liver, spleen, and kidney are the key immune organs of aquatic animals and the primary locations where immune genes function to prevent pathogen invasion. *P. sinensis* was found to have higher expression levels of *PsTLR2*, *PsTLR3*, and *PsTLR5* in the intestine, liver, spleen, and kidney than other tissues. This suggests that these tissues are the main sites for the synthesis and storage of the *PsTLR2*, *PsTLR3*, and *PsTLR5* genes in *P. sinensis*. Although TLR genes have different expression patterns in different species, they are widely expressed in different tissues and organs, indicating that they play important roles in the immune system [29,30,31,32,33].

Numerous investigations have demonstrated that *A. hydrophila* can infect *P. sinensis* and result in a range of illnesses [34,35,36]. TLRs are crucial PRRs that have the ability to identify germs and trigger the immune system. Previous findings revealed that TLR gene expression changed after *A. hydrophila* infection in many aquatic animals, such as *Danio rerio* [37], *Acipenser sinensis* [38], and *T. fulvidraco* [39]. *PsTLR2*, *PsTLR3*, and *PsTLR5* expression were also up-regulated following *A. hydrophila* infection in *P. sinensis*. TLR2 can recognize peptidoglycan, especially peptidoglycan from Gram-positive and Gram-negative bacteria, lipoproteins and lipopeptides, human cytomegalovirus (CMV) glycoprotein B, *Mycobacterium tuberculosis* antigens, and other small-molecule compounds [40]. TLR3 recognizes a variety of double-stranded RNA molecules, including viral dsRNA and synthetic dsRNA mimetics such as polyinosinic-polycytidylic acid (poly (I:C). In addition, TLR3 recognizes single-stranded RNAs (ssRNAs) and mRNAs, which may be derived from damaged host cells or tissues [41]. TLR5 is able to recognize flagellin from different bacterial species [42]. The expression of *PsTLR2* and *PsTLR5* was up-regulated in the early stage of infection, indicating that *PsTLR2* and *PsTLR5* recognized the peptidoglycan components and flagellin of invading *A. hydrophila* and triggered the immune response. However, *PsTLR3* mainly recognizes viral RNA molecules and cannot respond to the invasion of *A. hydrophila* at the early stage of infection. At the late stage of infection, *A. hydrophila* may be phagocytosed and decomposed by macrophages in vivo to produce RNA molecules, which are recognized by *PsTLR3* and begin to up-regulate its expression. This phenomenon suggests that TLRs play an important role in the immune process against bacterial invasion.

Analyzing the structure and function of proteins is crucial in post-genome research, and the most fundamental tool for this is an efficient protein expression system. Because *E. coli* expression systems are inexpensive and convenient, they are frequently employed to produce recombinant proteins [43,44,45]. Unfortunately, improper folding prevents the majority of recombinant proteins from forming soluble proteins [46]. In order to obtain more soluble target proteins, the cell can restrict the expression of other cellular proteins in the low-temperature environment by utilizing the CspA (cold shock protein A) promoter and related components found in the pCold-TF plasmid [47,48]. For instance, Hu used the pCold-TF vector to successfully express the recombinant protein BpEG01790 [49]. The zymography analysis along with SDS-PAGE verified that the recombinant protein was a soluble and active protein. Maya Uenobe used the pCold-TF prokaryotic expression method to successfully produce the soluble recombinant protein *ayu TNF* [50]. The pCold-TF expression system was also chosen for this investigation in order to create the recombinant plasmids pCold-TLR2-LRR, pCold-TLR3-LRR, and pCold-TLR5-LRR. Following IPTG induction, the recombinant plasmids successfully expressed the soluble proteins rPsTLR2, rPsTLR3, and rPsTLR5, as demonstrated by the SDS-PAGE results, which were in line with the anticipated outcomes. The acquisition of the proteins rPsTLR2, rPsTLR3, and rPsTLR5 gave rise to a solid foundation for additional research into the roles of *PsTLR2*, *PsTLR3*, and *PsTLR5*.

The primary pathogens that pose a threat to aquaculture include bacteria, fungi, and other microorganisms. Of particular concern are Gram-negative bacteria like *A. hydrophila* and *V. parahaemolyticus*, which have the potential to cause numerous diseases in aquatic animals and significantly impair aquaculture operations [34,35,36,51]. Numerous investigations have demonstrated the ability of TLR recombinant proteins to bind to harmful bacteria in vitro. For instance, Zhu discovered that TLR5 in Trachinotus ovatus could bind to *A. hydrophila*, *S. aureus*, *E. coli*, and *Vibrio anguillarum* [52]; Liu discovered that a recombinant protein of MaTLR14 from rice eel could bind to *A. hydrophila*, *E. tarda*, *S. aureus*, and Bacillus subtilis [53]. Wu discovered that TLR3 could attach itself to a variety of bacteria, including Photobacterium damselae, *E. coli*, *A. hydrophila*, *S. aureus*, and *Vibrio harveyi*, *Vibrio vulnificus*, and *Vibrio anguatus* [54]. The outcomes of this chapter demonstrated that *P. sinensis*’s recombinant proteins rPsTLR2, rPsTLR3, and rPsTLR5 could bind to microbes as well. Compared to rPsTLR2 and rPsTLR5, rPsTLR3’s capacity to bind to microorganisms was less potent at the same binding concentration. Gram-negative bacteria were more resistant to rPsTLR2’s binding than Gram-positive bacteria were, while fungi were more resistant to rPsTLR5’s binding than rPsTLR2. These findings demonstrated that *PsTLR2*, *PsTLR3*, and *PsTLR5* are capable of identifying microorganisms. They can also function as pattern recognition receptors to identify pathogens, transfer immunological data, and have a significant impact on the immune system’s reaction to antibiotics.

## Figures and Tables

**Table 1:** Primers used for cloning, qPCR expression, and plasmid construction of TLRs genes.

Primer Name	Primer Sequence (5′–3′)	Usage
PsTLR2-F	ATGAGATCAATAGGAAGCACTGGC	TLR2 cloning
PsTLR2-R	CTAGGATTTTAATGCTATTTTCAAATTAAA	TLR2 cloning
PsTLR3-F	ATGAGAGCTACCCTTTCCAGTTGG	TLR3 cloning
PsTLR3-R	TCAATGTATTTTGCTGCTAGATTTAAG	TLR3 cloning
PsTLR5-F	ATGTACTTCCCACAGTTTCTGCAG	TLR5 cloning
PsTLR5-R	CTACGAGATTGTCCTTATCGTTTGC	TLR5 cloning
PsTLR2-qF	TTCAGGCACTCAGATAACCACG	Real-time PCR
PsTLR2-qR	TTCTGCTGATTCTTAGGGACACC	Real-time PCR
PsTLR3-qF	GCACCACTTTGATAATGCTCCTC	Real-time PCR
PsTLR3-qR	CCCACTTCCTGTCCCTTCTTG	Real-time PCR
PsTLR5-qF	GATCCTGAGCATCACAATGTTACATCATC	Real-time PCR
PsTLR5-qR	GGCTACAACCATTATACCTGGCGATT	Real-time PCR
β-actin-qF	AGACCCGACAGACTACCTCA	Real-time PCR
β-actin-qR	CACCTGACCATCAGGCAACT	Real-time PCR
PsTLR2-LRRs-F	ggtatcgaaggtaggCATATGCCTTCAGGACTAACAACTGATGTCA	Plasmid construction
PsTLR2-LRRs-R	ctatctagactgcagGTCGACGTGACATTCAAACAGTGAAGCCG	Plasmid construction
PsTLR3-LRRs-F	ggtatcgaaggtaggCATATGACCTGCTCCTGGCTCTGTGTAA	Plasmid construction
PsTLR3-LRRs-R	ctatctagactgcagGTCGACTTTGCAGGGTGAAATGTCAAAA	Plasmid construction
PsTLR5-LRRs-F	ggtatcgaaggtaggCATATGCCAAACCTTCAAACCTTAGATTTAGG	Plasmid construction
PsTLR5-LRRs-R	ctatctagactgcagGTCGACATTACACCCATCAAGTGCCACTG	Plasmid construction

## Data Availability

The original contributions presented in the study are included in the article/Supplementary Materials, further inquiries can be directed to the corresponding author.

## Supplementary Materials

The following supporting information can be downloaded at: https://www.mdpi.com/article/10.3390/genes15091230/s1, Figure S1: Amino acid sequence alignment of PsTLR2 and XM_025185305.1 (“*” indicates sequence identity; “Spaces” indicate sequence inconsistencies; the length of the sequence is marked by the red box). Figure S2 Amino acid sequence alignment of PsTLR3 and XM_014576247.2 (“*” indicates sequence identity; “Space” and “Red triangle” indicate sequence inconsistencies; the length of the sequence is marked by the red box). Figure S3 Amino acid sequence alignment of PsTLR5 and XM_025180981.1 (“*” indicates sequence identity; “Spaces” indicate sequence inconsistencies; the length of the sequence is marked by the red box).
