# Supplementary material for: Toll-like Receptor Expression in Pelodiscus sinensis Reveals Differential Responses after Aeromonas hydrophila Infection
Source: Genes (Basel). 2024 Sep 20;15(9):1230. doi: 10.3390/genes15091230 (PMC11431187; doi:10.3390/genes15091230)
Supplement: Supplementary file 1 [file genes-15-01230-s001.zip › genes-3147004-supplementary.pdf]

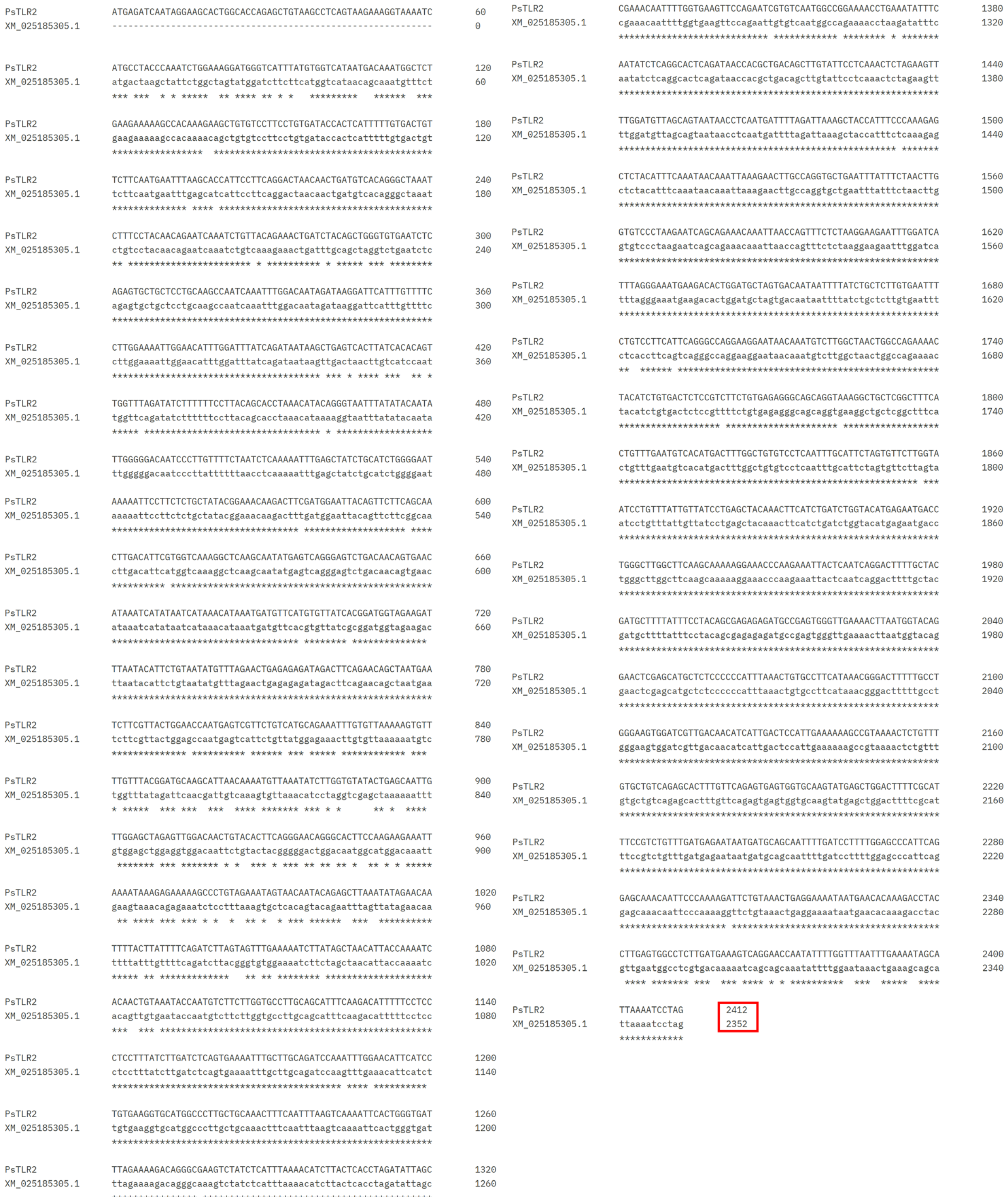

Figure S1 Amino acid sequence alignment of *PsTLR2* and XM_025185305.1(“*” indicates sequence identity; “Spaces” indicate sequence inconsistencies; the length of the sequence is marked by the red box.)

Note: Just the CDS region of XM_025185305.1 was selected for alignment.

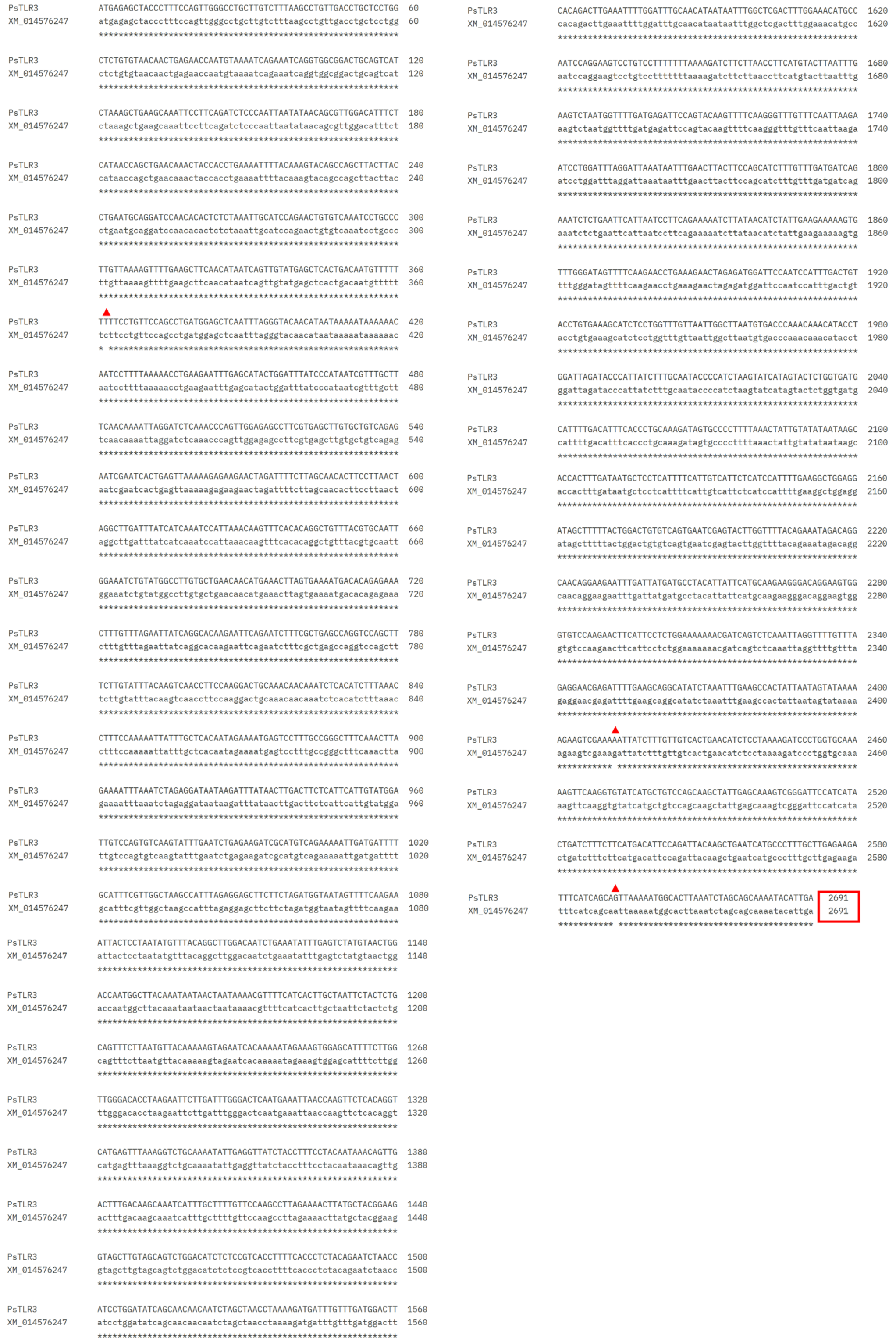

Figure S2 Amino acid sequence alignment of *PsTLR3* and XM_014576247.2 (“*” indicates sequence identity; “Space” and “Red triangle” indicate sequence inconsistencies; the length of the sequence is marked by the red box.)

Note: Just the CDS region of XM_014576247.2 was selected for alignment.

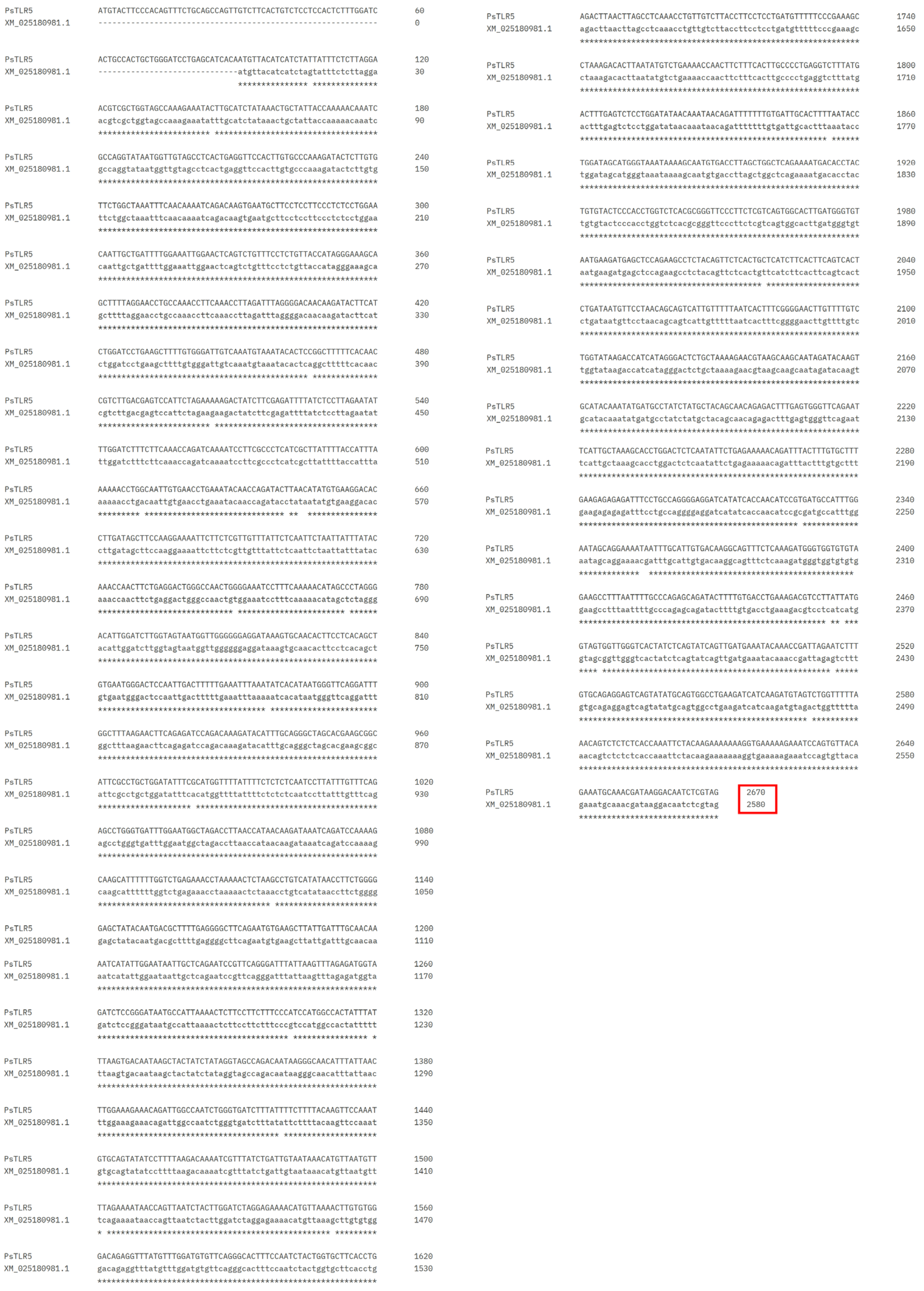

Figure S3 Amino acid sequence alignment of *PsTLR5* and XM_025180981.1 (“*” indicates sequence identity; “Spaces” indicate sequence inconsistencies; the length of the sequence is marked by the red box.)

Note: Just the CDS region of XM_025180981.1 was selected for alignment.
